# Gastroprotective Effects of *Periplaneta americana* L. Extract Against Ethanol-Induced Gastric Ulcer in Mice by Suppressing Apoptosis-Related Pathways

**DOI:** 10.3389/fphar.2021.798421

**Published:** 2021-12-15

**Authors:** Shu Fu, Jiamei Chen, Chen Zhang, Jinfeng Shi, Xin Nie, Yichen Hu, Chaomei Fu, Xiaofang Li, Jinming Zhang

**Affiliations:** ^1^ State Key Laboratory of Southwestern Chinese Medicine Resources, College of Pharmacy, Chengdu University of Traditional Chinese Medicine, Chengdu, China; ^2^ School of Pharmacy, Chengdu Medical College, Chengdu, China; ^3^ Key Laboratory of Coarse Cereal Processing, Ministry of Agriculture and Rural Affairs, Sichuan Engineering & Technology Research Center of Coarse Cereal Industralization, School of Food and Biological Engineering, Chengdu University, Chengdu, China

**Keywords:** *Periplaneta americana* L., gastric ulcer, MyD88/NF-κB, PAS staining, HPLC

## Abstract

Although *Periplaneta americana* L. and its modern preparation, Kangfuxin liquid, have been extensively applied for ulcerative diseases in gastrointestinal tract (e.g., gastric ulcer (GU) and ulcerative colitis, the effective components and potential mechanisms) remain unclear. In accordance with the accumulating research evidences, the relieving/exacerbating of GU is noticeably correlated to focal tissue programmed cell death. Herein, gastro-protective effects of the effective *Periplaneta americana* L. extract (PAE) fraction were assessed *in vitro* and *in vivo*, involving in programmed cell death-related signaling channels. To screen the effective PAE fraction exerting gastroprotective effects, several PAE fractions were gained based on a wide range of ethanol solution concentration, and they were assessed on ethanol-induced ulcer mice. Based on HPLC investigation with the use of nucleosides, the chemical composition of screened effective PAE, extracted by 20% ethanol, was analyzed in terms of quality control. Based on CCK-8 assay, the protective effects on GES-1 cells, impaired by ethanol, of PAE were assessed. After 3 days pre-treatment with PAE (200, 400, 800 mg/kg), the gastric lesions were assessed by tissue morphology, and periodic acid-schiff (PAS) staining, as well as hematoxylin and eosin (H&E) based histopathology-related investigation. The levels for inflammation cytokines (IL1-β, TNF-α, IL-18, PGE2, and IL-6), antioxidant indices (SOD and MDA) were examined via ELISA. In the meantime, based on Western Blotting assay, the expression levels of some programmed cell death-related protein targets (NLRP3, caspase-1, NF-κB p65, MyD88, and TLR4) were analyzed. As revealed from the results, PAE is capable of alleviating gastric mucosa impairment, suppressing the inflammatory cytokines, and down-regulating the MyD88/NF-κB channels. Accordingly, 20% ethanol extract of *Periplaneta americana* L. would contribute its gastroprotective effects, thereby providing the evidence that its anti-GU mechanisms correlated with inhibiting programmed cell death channel.

## Introduction

Gastric ulcer (GU) refers to a prevailing disorder relating to the stomach and intestines with effects on more populations globally (Boligon et al., 2014). GU popularity reaches 2.4% within west people as well as 6.1% within Chinese population (Xie et al., 2020). Though GU has disputed etiology and the origination and development of the disease, the disbalance assumption of destruction and offense factor pertaining to mucosal barriers was extensively recognized (Chen et al., 2015; Chen et al., 2018). Different elements are likely to facilitate the process of forming GU [e.g., nonsteroidal anti-inflammation drug (NSAID), consumption of alcohol, tobacco-smoking, *Helicobacter pylori* infection, and psychology stress (Adhikary et al., 2011)]. More and more research reported that programmed cell death is critical channels under the catalysis from caspases downstream pertaining to impairment of gastrointestinal mucosa epithelium (Ahmed et al., 2021). It acts via molecule signaling channels exhibiting the characteristics of the cell regulatory cycle’s initiating, mediating, executing and regulating processes. A recent research built correlations of apoptosis and autophagy to indomethacin-triggered mucosa erosion and ulceration (Gebril et al., 2020). Besides, mitochondrion channel mitigated programmed cell death had correlations to the act or process of causing or getting *H. pylori*. The act or process of causing or getting *H. pylori* up-regulated pro-apoptosis protein Bax, whereas it down-regulate anti-apoptosis protein Bcl-2 condition within gastric mucosa (Liu et al., 2005). It is noticeable that stimulation for caspase three and nine is reported within one gastric adenocarcinoma cell line for responding *H. pylori* culture (Zhang et al., 2007).

A number of drugs (e.g., H2 receptor antagonist drug, antacid drug, proton pump inhibiting drug (omeprazole) and antibiotic drug) can be used in terms of treating GU (Ren et al., 2020). Nevertheless, the mentioned medications encounter pivotal issues as impacted by their insufficient efficacy in resisting GU as well as noticeable side effect (e.g., cardiovascular disease risk, hypergastrinaemia, osteoporotic bone fracture, impotence, hypoacidity and gynecomastia) (Chakravarty and Gaur, 2019; Zhang et al., 2019a; Kulikova et al., 2020; Zhou et al., 2020). For this reason, novel drugs capable of being highly efficient and less toxic should be developed to prevent and treat GU.

Hence, the application of traditional Chinese medicine and their extracts turns out to be a hotspot of widespread concern. Clinically and experimentally related research reported their clinically related benefit and less side effect to treat GU (Bi et al., 2014). *Periplaneta americana* L. (PA) (Song et al., 2017), i.e., American cockroach, generally acts as crude drugs exhibiting the long clinically related use history. Given the relevant records within Shennong’s Classic of Materia Medica, the classics of traditional Chinese medicine (TCM), PA was primarily adopted for promoting circulations of blood, removing blood stasis in accordance with TCM (Ma et al., 2018). Kangfuxin (KFX), an extract of PA, gained the approval from China Food and Drug Administration (CFDA) in 1998, involving primarily small molecular peptide, amino acid and nucleotide (Lu et al., 2019). KFX was extensively applied in terms of healing of tissue wounds, particularly within gastric and duodenal ulcer (Chen et al., 2016). In accordance with increasing literature, KFX can exert gastroprotective influence through the decrease in oxidation stresses and endoplasmic reticulum stress in resisting gastric ulcer triggered by ethanol in rats (Chen et al., 2016; Shen et al., 2017). Considerable clinically related tests proved KFX to help cure GU, under the support from animals experiments (Zou et al., 2019).

On the whole, in accordance with the mentioned research, KFX refers to a therapeutic drug with high prospect to treat GU. However, the active substance composition and protective system pertaining to KFX to improve GU remain not clear. In accordance with existing reports, the anti-GU active parts of PA were screened and the quality of the effective parts was controlled. The anti-GU mechanism of *Periplaneta americana* L. was assessed from the perspective of inhibition of programmed cell death channel.

## Materials and Methods

### Materials

The reference standards uracil, hypoxanthine, inosine and gland pyrimidine were purchased from National Institute for Food and Drug Control (Beijing, China). Sucralfate was purchased from Abcam Co., Ltd. Gastric epithelial cell line (GES-1), Dulbecco’s modified Eagle’s Medium (DMEM), and fetal bovine serum (FBS) were purchased from Boster Biological Technology Co., Ltd., China) China. Mouse monoclonal anti-beta-actin and rabbit polyclonal anti-TLR4 were obtained from Multisciences BIOTECH, Co., Ltd. (Hangzhou, China). Rabbit polyclonal anti-NALP3/CIAS1 and rabbit polyclonal anti-Caspase1 p20 were obtained from Abcam (Cambridge, UK). Rabbit polyclonal anti-MyD88 was purchased from Boster Biological Technology Co. Ltd. (China). Rabbit polyclonal anti-NF-κB p65 was obtained from Servicebio, Technology Co., Ltd. (Wuhan, China).

### Preparation of PAEs With Several Concentrations of Ethanol

*Periplaneta americana* L. (PA) was primarily identified by microscopic and thin layer chromatography identification, according to Chinese Pharmacopoeia 2020 edition. 1 kg of dry PA was crashed into crude powder and degreased twice with petroleum ether (1:10) for 3 h every time, and dried by a vaccum drying method. Each of 100 g the degreased PA powder was extracted with 1000 ml of 20, 40, 60, and 80% ethanol through reflux extraction for 2 h. All extracts were collected and concentrated with one rotary evaporating device at 40°C. Subsequently, the extracts received the lyophilization after filtration. The yield of PAEs was calculated.

### Screening the Effective PAE Fraction on Ulcer Mice Triggered by Ethanol

Male Sprague-Dawley (200–250 g) mice were raised in the specific pathogen free (SPF) animal center at Chengdu University of TCM with one animal quarter under air conditioning with 12 h lightness/without light, temperature 22 ± 2°C and humidity 50 ± 10% cycle. The mice could freely have food and water. The animal experiments here were carried out based on the International Guideline recommendation in terms of using and caring experiment-related animal, as permitted by the Committee responsible for animal experiment ethics in Chengdu University of TCM.

We launched rat gastric mucosa impairment in accordance with our previous study. When the acclimatization was achieved for a week, the experiment mice were administrated with absolute ethanol 6 ml/kg animal and received the random classification to five groups (*n* = 6 per group). (1) Model group; (2) 20% ethanol extract (20 mg/kg) group; (3) 40% ethanol extract (20 mg/kg) group; (4) 60% ethanol extract (20 mg/kg) group; and (5) 80% ethanol extract (20 mg/kg) group. The mice were treated orally with PAE once a day for 14 consecutive days except for model cohorts which were administered saline. On the last day of the treatment, the experimental mice were deprived of food but allowed free access to water for 24 h after the final administration. Next, the animals were sacrificed, and the stomach tissues were removed, rinsed gently with cold saline and then photographed. The ulcer index in terms of square millimeters (mm^2^) and ulcer inhibition percentage (%) were achieved in accordance with the previous method with a slight change. In this method, the surface of the injured area was ascertained with a ruler, and the degree of the ulcer’s degree was determined based on the severity of the ulcer with the use of Table 1.

**TABLE 1:** Gastric ulcer scoring system based on the severity of ulcer.

Ulcer score	Gastric lesions
0	No lesion
1	Mucosal edema and petechiae
2	One to five small lesions (1–2 mm)
3	More than five small lesions or one intermediate lesion (3–4 mm)
4	Two to more intermediate lesions or one gross lesion (>4 mm)
5	Perforated ulcers

### HPLC Investigation

HPLC investigation was performed for PAE, together with reference compounds uracil, hypoxanthine, inosine and gland pyrimidine based on high performance liquid chromatography (HPLC) System (LC-2030C, SHIMADZU, Japan) with an auto-injector sampler programmed at 10 μL, with the use of a C_18_ column (Agilent five HC-C_18_ 250 × 4.6 mm) at a column temperature of 25°C. All operations, acquisitions and data investigation were regulated with the use of the Chemstation software (SHIMADZU, Japan). The mobile phase was composed by A (methanol: water: acetic acid = 30:69.8:0.2, v/v) and B (methanol: water = 1:1, v/v). Samples were eluted in accordance with the following gradient: 0–10 min 100% A isocratic, 10–20 min 100–70% A, 20–21 min 70–50% A, 21–22 min 50–100% A, 22–25 min 100% A isocratic. The flow rate and the optimized detection wavelength reached 0.6 ml/min and 254 nm, respectively.

### Cell Culture

We incubated human gastric epithelial cell line (GES-1) in cell culture medium (CCM) covering 89% Dulbecco’s modified Eagle’s Medium (DMEM), 10% fetal bovine serum (FBS), 1% antibiotic antimycotic solution. Then, the culture was performed at 5% CO_2_, 37°C.

### Gastroprotective Effects of PAE *In Vitro*

The effects of PAE on the proliferation of GES-1 cells after 24 and 48 h treatment were assessed. To specific, the GES-1 cells were seeded in a 96-well plate with the density of 6 × 10^3^ cells per well to adhere for 24 h, and then treated with PAE at several concentrations (5, 10, 20, 40, and 80 μg/ml) for 24 and 48 h, respectively. CCK-8 assay was performed to assess the proliferation of GES-1 cells with PAE.

To assess the gastro-protective effects of PAE on cell impairment model under the cause of ethanol, the GES-1 cells treated with 100 μM ethanol was employed. Several concentrations of PAE were treated with the mentioned cells. Briefly, 6.0 × 10^3^ cells/well were cultured in 96-well plates for 16 h. And then, the mentioned cells were divided into the control (medium), model (medium + ethanol) and PAE treatment cohorts with several concentrations of PAE (5, 10, 20, 40, and 80 μg/ml). After 24 h culture, the CCK-8 assay tool was employed to assess cell viability.

### Gastroprotective Effect on Ulcer Mice Triggered by Ethanol *in vivo*

The ICR mice (6–8 weeks of age) were purchased from SPF (Beijing) Biotechnology Co. Ltd. (Chengdu, China). Mice were housed in collective cages at 25 ± 1°C with free access to laboratory chow and distilled water. The animal experiments were approved by the ethics committee of the Chengdu University of Traditional Chinese Medicine (CDUTCM, permit SYXK (Chuan) 2020-124), and all animal experiments were conducted in strict accordance with the Guidelines for the Care and Use of Laboratory Animals of the Ministry of Science and Technology of China.

After 3 days of adaptive feeding, the mice were randomly divided into six cohorts: normal control cohort (saline), gastric ulcer model cohort (saline + ethanol) and positive control cohort (200 mg/kg of sucralfate), PAE-L cohort (200 mg/kg of PAE), PAE-M cohort (400 mg/kg of PAE), and PAE-H cohort (800 mg/kg of PAE). All drugs were pre-administrated intragastrically once daily for 3 days before induction. On the third day, following a 24 h fast with free access to water, except normal control cohort, all mice were administrated intragastrically with absolute ethanol 6 ml/kg animal. After 2 weeks, the animals were sacrificed, and the stomach tissues were removed, then rinsed gently with cold saline and photographed. To measure the gastric lesions, the lesioned gastric area rate was determined with the use of the program Image J (Zhang et al., 2019b).

### Histopathological Investigation

For histopathological investigation, gastric tissues fixed in 4% paraformaldehyde were embedded in paraffin and then cut into 5 mm thick sections. The mentioned tissue sections were stained with hematoxylin and eosin (H&E), periodic acid-schiff (PAS). Under a light microscope (Olympus, Tokyo, Japan), an assessment was blindly conducted on the sections by an experienced and board-certified pathologist in accordance with the criteria (Zhang et al., 2021).

### Determination of Inflammatory Cytokines

The gastric tissue segments were homogenized with nine folds of cold PBS, centrifuged for 15 min at 6000 rpm/min, and the supernatant was collected. The amounts of MPO, SOD, IL1-β, IL-6, IL-18, PGE2, and TNF-α in gastric homogenate were achieved with the use of the commercial detection tools.

### Western Blotting

Homogenized gastric tissue was lysed with radioimmunoprecipitation assay (RIPA) lysis buffer (Beyotime, Nanjing, China) covering protease and phosphatase inhibitors, and centrifuged at 12,000 rpm for 20 min at 4°C. The overall protein concentration was ascertained with the use of a BCA protein assay tool in the light of the manufacturers’ instructions. Commensurable protein was resolved by 10% sodium dodecyl sulfate-polyacrylamide gels and subsequently transferred to polyvinylidene fluoride membrane (Xu et al., 2021). The blots were blocked for 2 h with 5% skim milk in TBST buffer, followed by incubation throughout the night under the temperature of 4°C based on primary antibody: mouse monoclonal anti-beta-actin (1:3,000), rabbit polyclonal anti-NALP3/CIAS1(1:1,000), rabbit polyclonal anti-Caspase1 p20 (1:1,000, rabbit polyclonal anti-TLR4 (1:1,000), rabbit polyclonal anti-MyD88 (1:1,000) and rabbit polyclonal anti-NF-κB p65 (1:1,000). Subsequently, the washing was achieved for immunoblots by employing TBST 3 times, and the probing process was achieved by adopting anti-rabbit horseradish peroxidase-conjugated secondary antibody (1:15,000 dilution in TBST) under the ambient temperature for 2 h. Next, the detection was achieved for immunoreactive protein with the use of an optimized system for chemiluminescence western blotting detection (GE Health Care; Little Chalfont, Buckinghamshire, UK). The immunoblotting tests were performed 3 times.

### Statistical Investigation

All data had the expression of mean ± standard deviation (SD). Statistical investigation was carried out with the use of SPSS 17.0 statistical software with One-way ANOVA or Kruskal-Wallis H-test and all charts were drawn by Graphpad Prism 5.0 (Graphpad Software, San Diego, USA). *p*-values of <0.05 had statistical significance.

## Results

### Bioactive Fraction Screening

**FIGURE 1:**
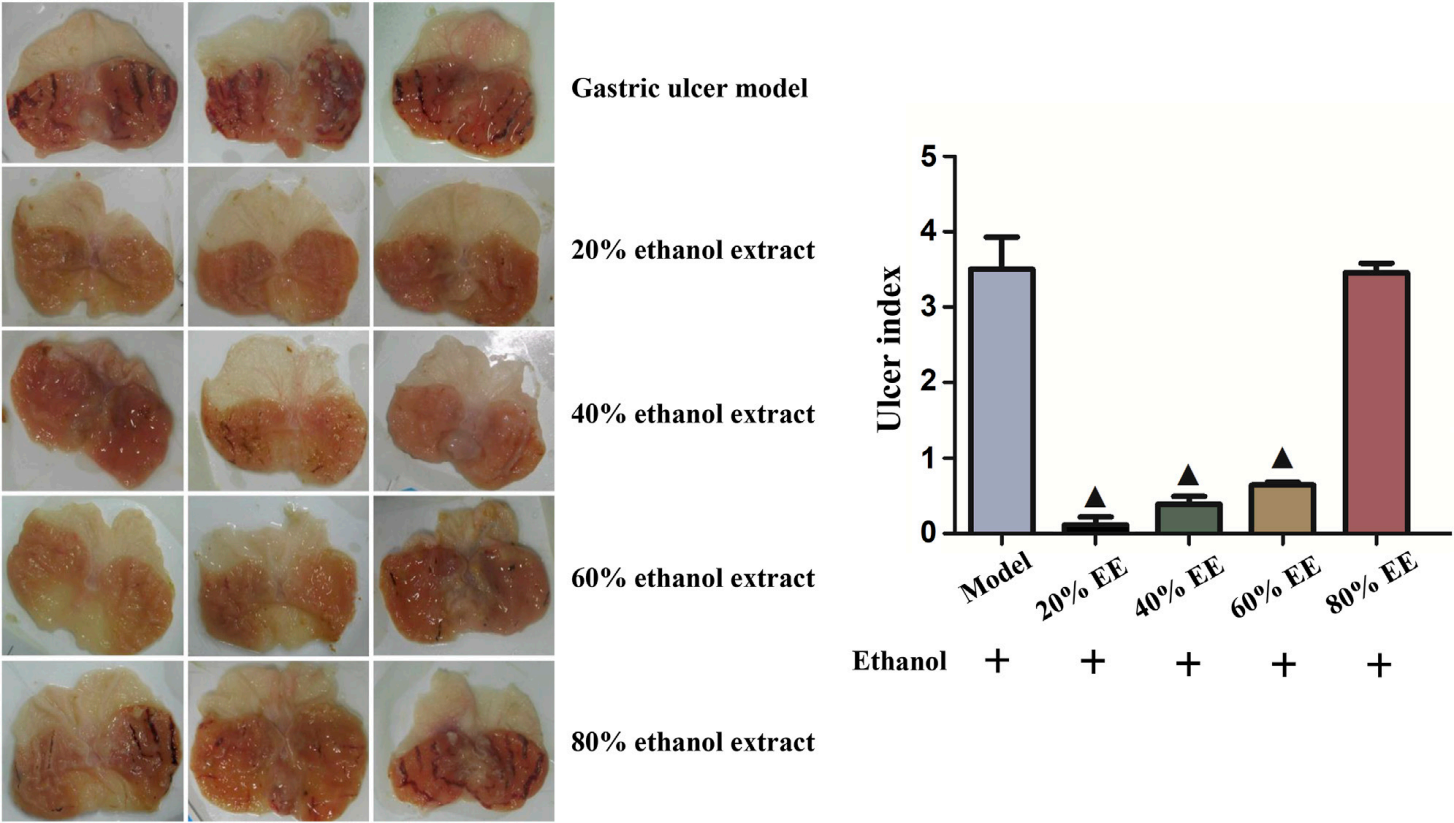
Effects of PAE extracted by a series of concentration of ethanol on the gross appearance of the gastric mucosa and ulcer index in ethanol-induced gastric ulcer in rats. ^▲^ *p* < 0.05, PAE treatment group vs. ethanol-induced group.

Acute gastric lesion was triggered through administrating ethanol in intra-gastric. According to Figure 1, we found a significantly broadening area of the formation of gastric ulcer within ethanol-stimulated mice. Treatment with PAE with several ethanol extraction processes achieved different therapeutic efficacies. In the gastric ulcer triggered by ethanol model cohort, mucosa tissues were extensively destroyed and necrotic, exhibiting severe hemorrhage, edema and leukocyte infiltration. The 20% EE, 40% EE, and 60% EE could effectively prevent the severe gastric mucosa impairment attributed to ethanol. In particular, mice pretreated with 20% EE before ethanol intoxication exhibited good protective effect against tissue impairment by ethanol, in which mucosa color was normally pink while not being thickened, also hemorrhages and congestion were not identified. The ulcer index score of ethanol treatment cohort rose to 3.59, whereas pretreatment of 20% EE, 40% EE, and 60% EE significantly (*p* < 0.05) decreased the UI scores in comparison with the ethanol treatment cohort. The ulcer index of 20% EE was lowest. Almost none of macroscopic ulcer or hemorrhagic spot could be found. Coupled with the high yield of 20% EE (36.7 ± 6.2%), therefore, we employed the 20% EE fraction for the subsequent experiments.

### Chemical Composition of PAE (20% EE)

**FIGURE 2:**
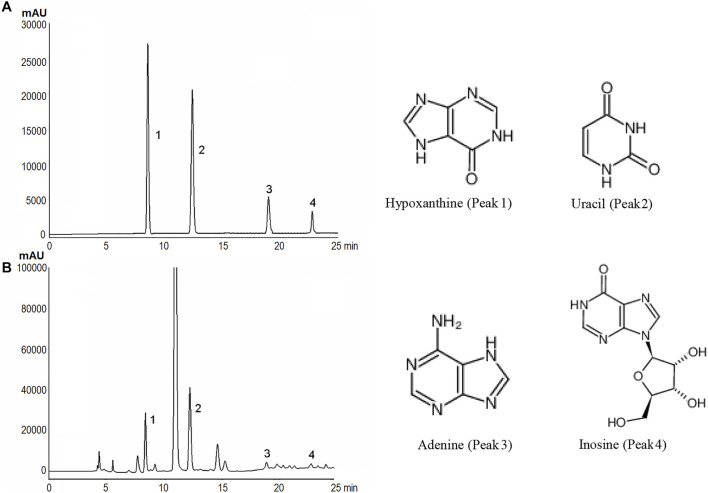
HPLC spectra and chemical structures of these representative markers of PAE obtained with 20% ethanol extraction. **(A, B)** represented the chromatogarm of four representative markers and PAE.

According to Figure 2, an analysis was conducted on four standard substances under the mixture, and a satisfied separation degree and methodology study was acquired. The normal curve and linear scope of the mentioned compounds included: hypoxanthine: y = 312163.70x + 103.22 (*r* ^2^ = 0.999992, 0.11–0.44 mg/ml), uracil: y = 1110949x + 371 (*r* ^2^ = 0.999991, 0.12 mg/ml∼0.49 mg/ml), adenine: y = 909935x + 3705 (*r* ^2^ = 0.9998, 0.039–0.12 mg/ml) and inosine: y = 75123x − 24 (*r* ^2^ = 0.99995, 0.18–0.75 mg/ml). Accordingly, we found four compounds, i.e., hypoxanthine, uracil, adenine and inosine, in PAE through the contrast with the retention time (t_R_) and UV spectrum of the relevant peaks within mixed standard solution. Their contents in PAE were achieved based on the external standard approach as 0.54, 1.21, 0.20, 0.87 mg/g, respectively.

### PAE Promoted GES-1 Cells Proliferation

**FIGURE 3:**
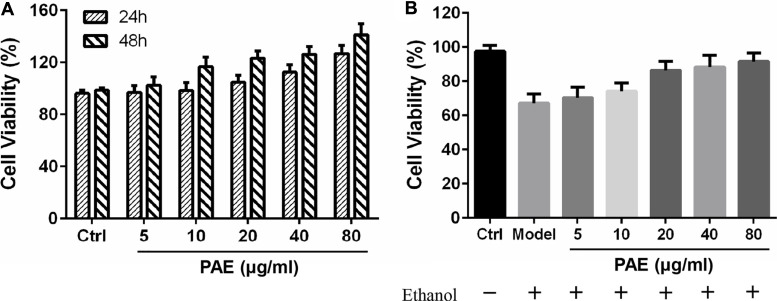
Effects of PAE on the proliferation of GES-1 cells. **(A)** The pretreatment of PAE promoted cell viability with 24 and 48 h treatment **(B)** Cell viability in the presence or absence of 100 μM ethanol for 24 h treatment.

For the assessment of the effect exerted by PAE on the cytotoxicity and proliferating process of normal GES-1 and ethanol-impaired GES-1 cells, we carried out the treatment for cells by using PAE (5–80 μg/ml) under 100 μM ethanol or not. After 24 and 48 h of treatment with PAE in normal GES-1 cells, GES-1 cells’ proliferation was promoted in a dose- & time- dependent manner (Figure 3A). This data indicated that PAE had no cytotoxicity and could facilitate the proliferation of epithelial cells. Additionally, the treatment with 100 μM ethanol would alter the appearance of GES-1 cells. The GES-1 cells treated by ethanol exhibited the poor cell adherence. Cytoplasm of the mentioned cells was shrunk, indicated the cytotoxicity and early programmed cell death. Some suspended cells could be found in culture medium. In accordance with CCK-8 assay, the cell viability of GES-1 cells treated by 100 μM ethanol was remarkably dropped (Figure 3B). However, the concentrations of 5, 10, 20, 40, and 80 μg/ml of PAE boosted GES-1 cell proliferation, in contrast with the reduced cell proliferation of GES-1 injured by ethanol. This implied that PAE was likely to facilitate GES-1 cell proliferation and protect the cell impairment by ethanol.

### Protective Effect of PAE Against Gastric Ulcer Triggered by Ethanol *In Vivo*

**FIGURE 4:**
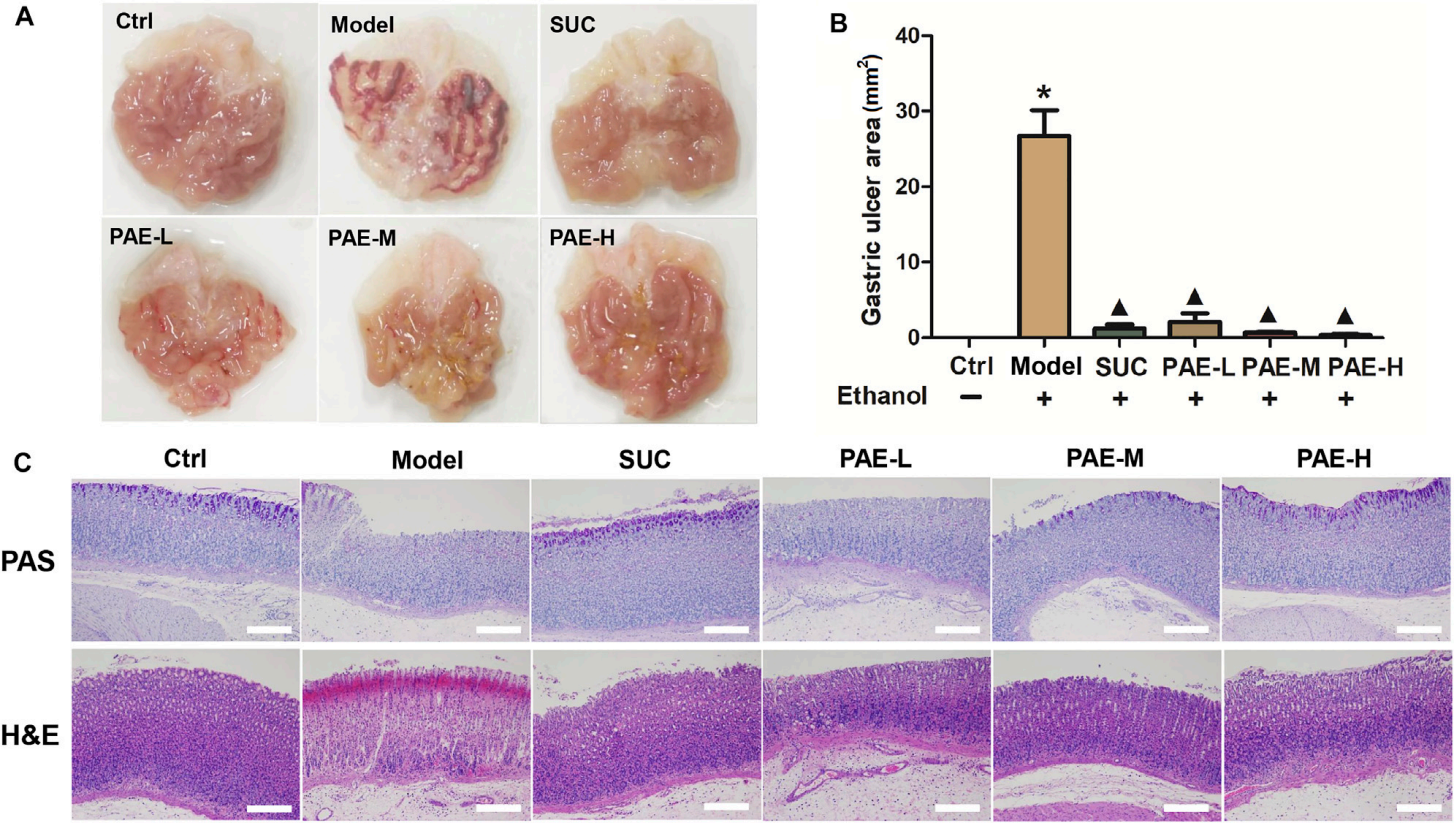
**(A)** Macroscopic view of the gastric tissue in gastric ulcer triggered by ethanol mice after administration of PAE. **(B)** Gastric ulcer area in each cohort. *p < 0.05, Ctrl group vs. Gastric ulcer model group; ^▲^ *p* < 0.05, PAE/SUC treatment group vs. Gastric ulcer model group; **(C)** Representative photomicrographs of sections from gastric wall samples by PAS and H&E staining.

Representative of images of the mentioned split stomachs were shown in Figure 4A. We reported significant tissue impairment and visible haemorrhagic mucosa lesions within the stomachs of the ethanol treatment mice, while the mice under the saline treatment indicated no lesion within gastric mucosa. As opposed to the mentioned, the administration of either SUC or PAE, particularly at the dosage of 400 mg/kg and 800 mg/kg PAE, would relieve the ulcer effectively. At the assistance of vernier caliper, the gastric ulcer area in stomach tissue was counted. According to Figure 4B, the ulcer area reached 27.6 ± 5.8 mm^2^ in the ethanol induce model cohort; pretreatment with PAE (200, 400, and 800 mg/kg) and 200 mg/kg of sucralfate efficiently attenuated the mentioned abnormal varieties, which was dependent of concentrations.

Histological changes in the stomach mucosa of the ulcer mice were visualized by PAS and H&E staining (Figure 4C). To specific, the change of mucin depletion in the inner membrane of mucosa was scored by PAS staining assay. As shown, the degree of purplish red on the surface of gastric tissue of gastric ulcer model cohort was much reduced in contrast with normal control cohort, indicated that the lower mucin content in ulcer cohort. Nevertheless, the treatment of SUC and PAE could relieve the mucin depletion. In particular, after administration of SUC and high dose of PAE (800 mg/kg), the positive rates of PAS staining were significantly augmented, which exhibited the similar level as normal control cohort. Histological assessment by H&E staining suggested more severe erosion of the gastric mucosa with hemorrhagic lesions extending deeply into the mucosa and submucosa, as well as extensive edema and leukocyte infiltration of the submucosal layer in ulcer cohort. However, the ulcerated mice pretreated with SUC and PAE exhibited less mucosa impairment in comparison with the ulcerated model mice, also complying with the remission of the ulcer area, edema, and leukocyte infiltration (Figure 4A).

### Levels of MPO and SOD in Gastric Tissue

**FIGURE 5:**
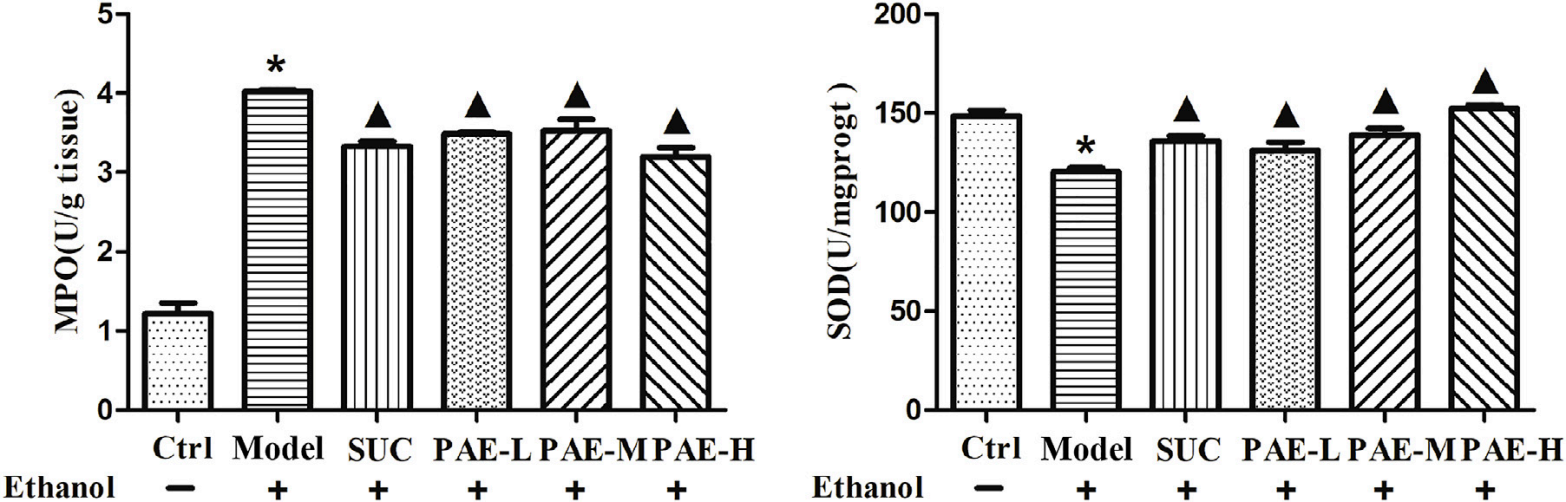
Effect of PAE on the levels of MPO and SOD in ethanol-stimulated gastric tissues. *p < 0.05, Ctrl group vs. Gastric ulcer model group; ^▲^ *p* < 0.05, PAE/SUC treatment group vs. Gastric ulcer model group.

MPO, as the indicator of neutrophils stimulation, is commonly used to assess the inflammation reaction. According to Figure 5A, in comparison with the control cohort, ulcer model cohort under the cause of ethanol significantly increased the levels of MPO (*p* < 0.05). After treatment of SUC and PAE at low, middle, and high dosage, the elevated level of MPO could be significantly dropped (*p* < 0.05), suggesting the alleviative inflammation in the mentioned treatment cohorts. Given oxygen free radicals involved in the occurrence and development of mucosa lesions, SOD is critical to preventing and treating gastric mucosa impairment and ulceration. According to Figure 5B, the level of SOD in ulcer mice triggered by ethanol were remarkably attenuated, in contrast with control cohort. Pretreatment with both SUC and PAE significantly increased the gastric SOD level (*p* < 0.05).

### Levels of Inflammatory Cytokines in Gastric Tissue

**FIGURE 6:**
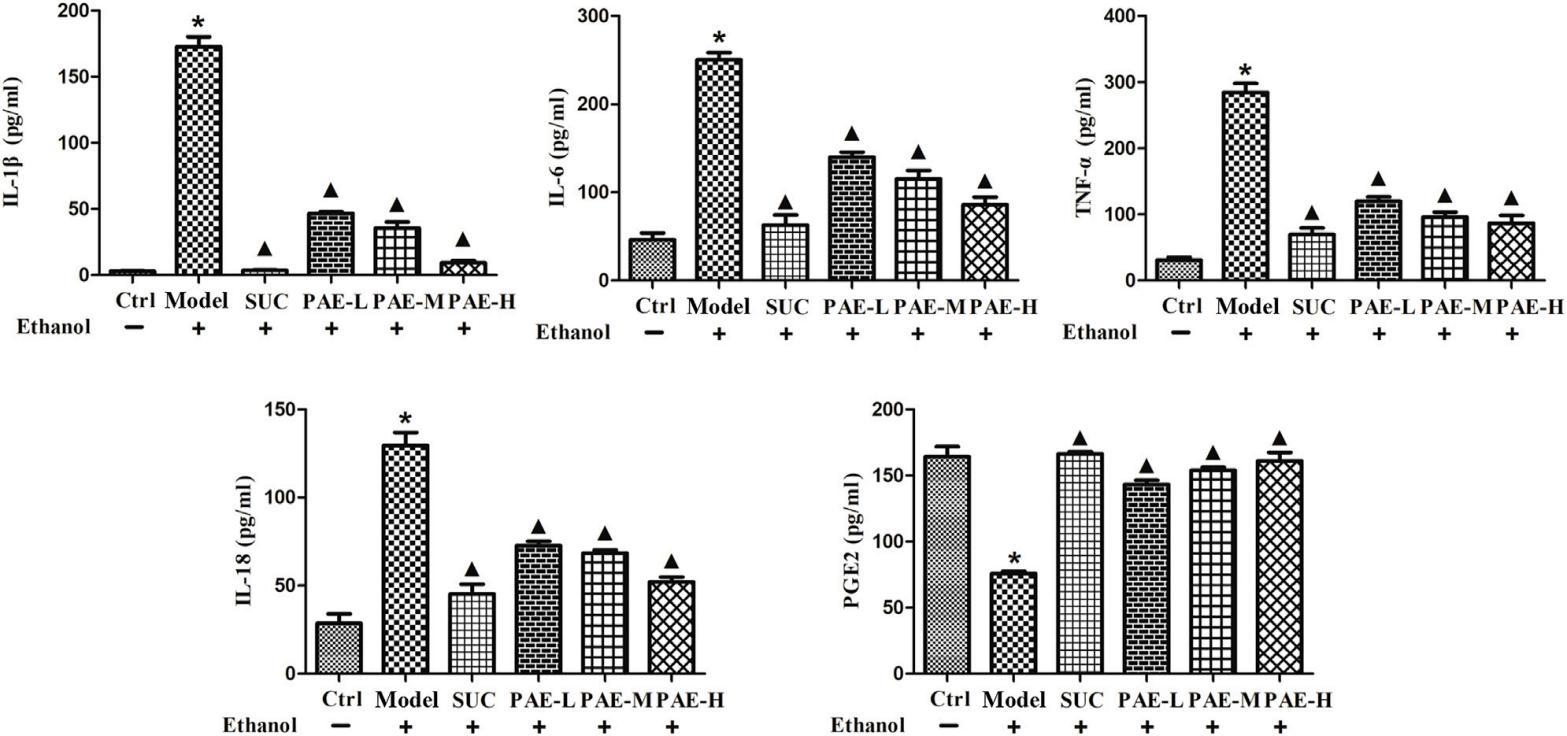
Effect of PAE on the levels of inflammatory cytokines, i.e., IL-1β, IL-6, TNF-α, IL-18, and PGE2, in ethanol-stimulated gastric tissues. *p < 0.05, Ctrl group vs. Gastric ulcer model group; ^▲^ *p* < 0.05, PAE/SUC treatment group vs. Gastric ulcer model group.

Inflammatory response is an inevitable mitigated factor in gastric ulcer triggered by ethanol, as commonly evidenced by the increased expression of pro-inflammatory cytokines. According to Figure 6, ethanol inducement would significantly increase the gastric pro-inflammatory cytokines (IL-1β, IL-6, TNF-α, IL-18). In contrast with control cohort, the amounts of IL-1β, IL-6, TNF-α, IL-18 in ulcer model cohort increased to 8.52-, 5.24-, 7.86-, and 4.16-folds, respectively. However, SUC and PAE could noticeably reverse this overproduction (*p* < 0.05). The down-regulation efficacy of PAE on pro-inflammatory cytokines exhibited a dose-dependent manner. The administration of high dose of PAE shown the strongest anti-inflammatory effects.

In addition, prostaglandin E2 (PGE2) was proven as a crucial mediator for sustaining the integrity of gastric mucosa defense and for gastric ulcer healing. PGE2 would control gastric acid secretion, stabilize mast cell membrane and stimulate tissue repair process, thereby critically impacting ulcer healing. The previous study revealed that the decreased level of PGE2 at the gastric mucosa would be involved in gastric ulceration and aggravate precancerosis. Thus, ethanol inducement could dramatically reduce PGE2, in contrast with normal control cohort, while either SUC or PAE significantly modulated the PGE2 level in the gastric homogenate in comparison with ulcer model cohort under the cause of ethanol (*p* < 0.05). The mentioned results suggested that PAE could prominently suppress the inflammatory response in stomach.

### Effects on Programmed Cell Death-Related Signal Channel

**FIGURE 7:**
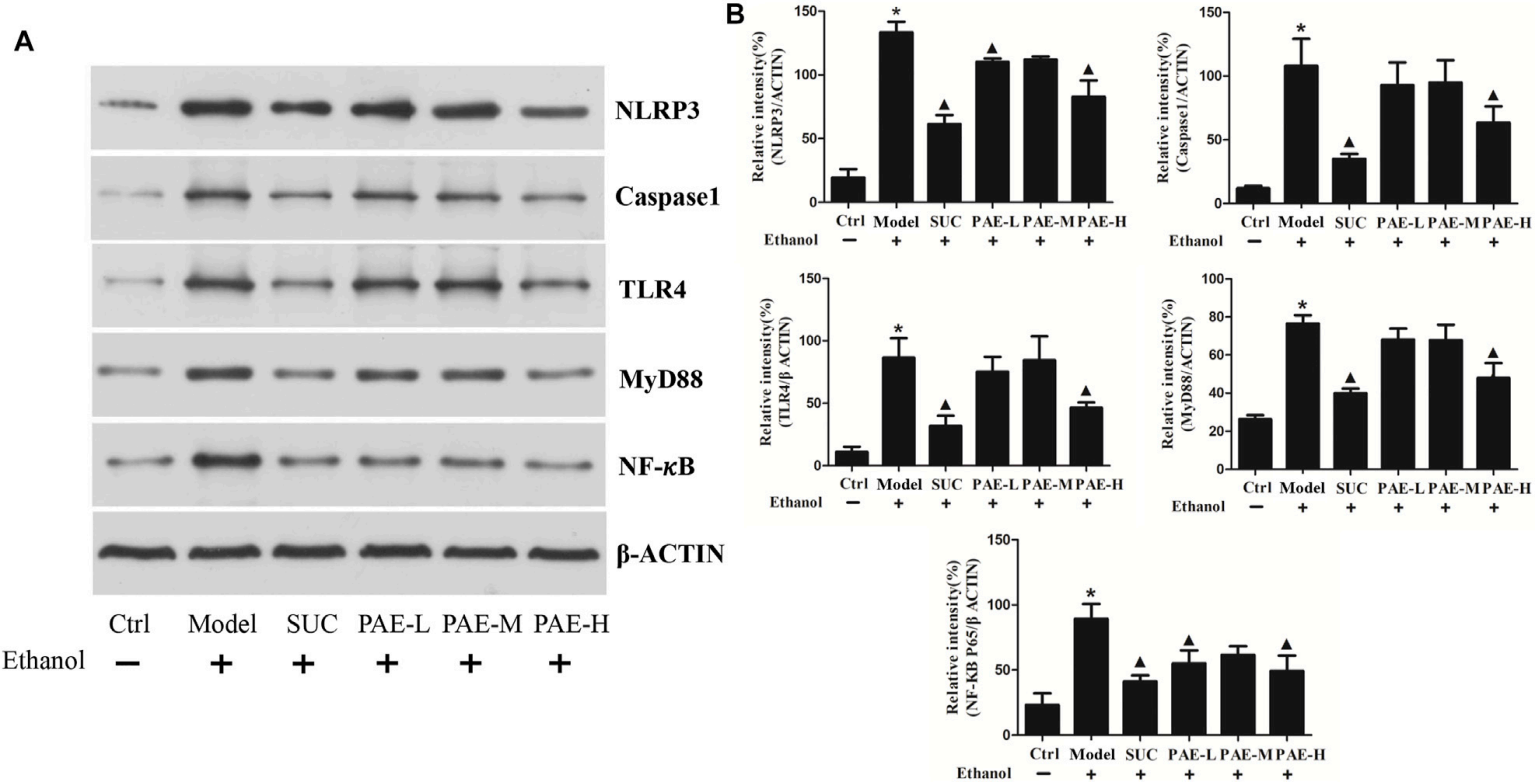
Influence of PAE on the expressions of programmed cell death-related proteins in ethanol-stimulated gastric tissues by western blotting. *p < 0.05, Ctrl group vs. Gastric ulcer model group; ^▲^ *p* < 0.05, PAE/SUC treatment group vs. Gastric ulcer model group.

The inflammasome, part of innate immunity and inflammation response, is critical to gastric impairment’s pathogenesis and progression under the cause of ethanol. It was acknowledged that to suppress the activity of nucleotide-binding domain-like receptor family pyrin domain-covering 3 (NLRP3) is an effective mechanism in resisting gastric mucosa impairment under the cause of ethanol. Herein, the NLRP3/caspase-1 cascade was examined mitigated by detecting the protein expression of NLRP3 and caspase-1 by western blotting assay. According to Figure 7, ethanol administration triggered the stimulation of the NLRP3/caspase-1 channel as demonstrated by 4.6-fold increase of NLRP3, and 4.12-fold increase of caspase-1, respectively, versus the control cohort. The stimulation of the NLRP3/caspase-1 channel was lowered by PAE administration, according to down-regulated protein expressions of NLRP3 and Caspase-1 levels by 38.6 and 41.2%, respectively in high dose of PAE cohort.

Moreover, the effects on the expressions of TLR4, MyD88, NF-κB P65 were achieved by western blotting assay. As illustrated, ethanol significantly up-regulated the expressions of the mentioned programmed cell death-related proteins, i.e., TLR4, MyD88, NF-κB P65. Nevertheless, pretreatment with PAE (800 mg/kg) and SUC effectively down-regulated the mentioned protein expressions. The mentioned findings denote that inhibition of the NLRP3/caspase-1 and NF-κB channels, at least partially, facilitates he ameliorative effects of PAE against gastric ulcer triggered by ethanol.

## Discussion

Researchers have widely used gastric ulcer model triggered by ethanol to study various promising agents for antiulcer treatment (Liu et al., 2012). One reason is that high alcohol consumption was considered as one of the most prevalent causes of GU in humans. It was proven that ethanol at high concentrations is capable of directly eroding gastric mucosa in 30–60 min, leading to gastric mucosa lesion and upper gastrointestinal bleeding (UGIB) (Liang et al., 2018). Moreover, ethanol induced GU model has the advantage over other GU models, such as pylorus ligation and stress (Ren et al., 2021). Through excessive oral administration of absolute ethanol in animal models, mucous membrane can be solubilized and then exposed to the proteolytic and hydrolytic actions of hydrochloric acid and pepsin, thereby causing gastric lesions. The mentioned lesions exhibit infiltration of inflammatory cell, desquamation of epithelial cell, hemorrhage and extensive submucosal edema, thereby resembling considerable characteristics exhibited by acute human peptic ulcer disease (Mousa et al., 2019). However, the underlying mechanisms of gastric ulcer triggered by ethanol are complex and still controversial. Many research cohorts have reported that the pathology of gastric ulcer triggered by ethanol generally involves three dimensions: oxidative stress, programmed cell death and inflammatory response (Liang et al., 2018; Badr et al., 2019; Zhou et al., 2020).

Notably, increasing evidence has shown that programmed cell death plays a key role in the gastrointestinal mucosa epithelial impairment. Gastroprotective effect of chlorogenic acid (Ahmed et al., 2021), gallic acid, patchoulene epoxide (Liang et al., 2018), and silymarin (Arafa Keshk et al., 2017) against GU in mice involves the modulation of programmed cell death signaling channel. Besides, programmed cell death cascade is closely correlated to oxidative stress and inflammatory response. To specific, overexposure to ROS and TNF-α can trigger caspase-dependent programmed cell death (Abdelwahab, 2013). Therefore, products having a strong apoptosis-resisting activity, with additional anti-inflammatory, and/or antioxidative properties, may represent potential candidates for GU therapy. Recent studies have demonstrated the inextricable link between NLRP3 inflammasome stimulation and cell death, covering ferroptosis, necroptosis, programmed cell death, as well as pyroptosis (Zheng and Kanneganti, 2020). Considerable cell death effectors (e.g., caspase-8, c-FLIP, GPX4 and MLKL) were confirmed to regulate inflammasome NLRP3 directly or indirectly stimulation and IL-1β release in response to a wide range of impairment-associated molecular patterns (DAMPs) and pathogen-associated molecular pattern molecules (PAMPs) (He et al., 2016).

We reported the cytoprotective, antiulcer, anti-inflammatory, antioxidant and apoptosis-resisting action of PAE in resisting GU under the cause of ethanol within SD mice initially. Besides, PAE is capable of noticeably rising programmed cell death-related protein (P65, NF-KB, MyD88, and TLR4) within gastric tissue under the cause of ethanol. Besides, when cells undergo oxidative stress under the cause of ethanol, they can induce and activate the release of the inflammation-associated factor NLRP3 inflammasome, activate Caspase-1 and eventually mature the effector pro-inflammatory cytokines (Hou et al., 2021). Our study has showed that PAE administration is capable of lowering the stimulation of the NLRP3/caspase-1 channel triggered by ethanol administration, while impacting inflammation and oxidative stress by regulating pro-inflammatory cytokines (IL-18, TNF-α, IL-6, IL-1β) and oxidative stress factors (MPO and SOD). Our result is also consistent with previous study. On the whole, we show vital evidences in relation to the noticeable anti-ulcer efficacy of PAE in resisting GU under the cause of ethanol on the basis of significant apoptosis-resisting activity.

## Conclusion

20% ethanol extract of *Periplaneta americana* L. exhibits significant gastroprotective effects against ethanol impairment *in vitro* and *in vivo*, indicating that this extract possessed a good potential as the anti-gastric ulcer agent. Pretreatment of *Periplaneta americana* L. extract could play a remarkable protective role on gastric tissue programmed cell death induced by ethanol, mitigated by MyD88/NF-κB signaling channels.

## Data Availability

The raw data supporting the conclusion of this article will be made available by the authors, without undue reservation.
